# In utero phthalate effects in the female rat: A model for MRKH syndrome✩

**DOI:** 10.1016/j.toxlet.2013.03.021

**Published:** 2013-03-28

**Authors:** Bethany R. Hannas, Kembra L. Howdeshell, Johnathan Furr, L. Earl Gray

**Affiliations:** a Reproductive Toxicology Branch, Toxicology Assessment Division, National Health and Environmental Effects Research Laboratories, Office of Research and Development, US EPA, Research Triangle Park, NC, United States; b Office of Health Assessment and Translation, National Toxicology Program, NIEHS, Research Triangle Park, NC, United States

**Keywords:** Reproductive malformations, Uterine, Critical period

## Abstract

Mayer–Rokitansky–Kuster–Hauser (MRKH) syndrome is characterized by uterine and vaginal canal aplasia in normal karyotype human females and is a syndrome with poorly defined etiology. Reproductive toxicity of phthalate esters (PEs) occurs in rat offspring exposed in utero, a phenomenon that is better studied in male offspring than females. The current study reports female reproductive tract malformations in the Sprague–Dawley rat similar to those characteristic of MRKH syndrome, following in utero exposure to a mixture of 5 PEs. We determined that females are ~2-fold less sensitive to the effects of the 5-PE mixture than males for reproductive tract malformations. We were not fully successful in defining the critical exposure period for females; however, incidence of malformations was 88% following dosing from GD8 to 19 versus 22% and 0% for GD8–13 and GD14–19, respectively. Overall, this study provides valuable information regarding female vulnerability to in utero phthalate exposure and further characterizes a potential model for the human MRKH syndrome.

## 1. Introduction

The widely used plasticizer compounds, phthalate esters, are recognized as reproductive toxicants in the male rat, with fetal exposure during the sexual differentiation period resulting in reproductive tract malformations reminiscent of human testicular dysgenesis syndrome (Fisher et al., 2003; Mylchreest et al., 1999; Sharpe and Irvine, 2004). Numerous studies focus on male reproductive toxicity of phthalates, whereas very little data is available on phthalate effects in female offspring exposed in utero. A small number of studies report female reproductive tract malformations following in utero exposure to the individual anti-androgenic phthalates, dibutyl phthalate (DBP) and diethylhexyl phthalate (DEHP) (Gray et al., 1999; Mylchreest et al., 1998); however, the percent incidence in these studies for any detected malformation (absence of vaginal opening, lack of vagina and partial or complete agenesis of the uterine horn(s)) was quite low.

We previously reported the ability to use individual phthalate potencies to predict the effects of a mixture of five phthalates on fetal male testicular androgens following in utero exposure (Howdeshell et al., 2008). The dosing scheme of the current postnatal investigation was designed as a follow-up to that fetal study. Although the postnatal study was designed to test the hypothesis that the postnatal effects following in utero exposure the same mixture correlate with the fetal effects in the male rat (Howdeshell et al., 2008), we serendipitously discovered extreme reproductive malformations in the female offspring. Therefore, the first study in the current investigation reports the reproductive effects on the females. Adult male reproductive malformations are being reported elsewhere (Howdeshell et al., in preparation). Furthermore, the second study of the current investigation was designed to define the critical exposure periods for inducing female reproductive tract malformations.

Previously, we found that several female rat offspring of dams exposed from weaning, through puberty, mating and pregnancy to 250 and 500 mg DBP/kg/d (oral gavage) displayed uterine malformations and reduced fecundity (Gray et al., 1999). Mylchreest et al. (1998) noted vaginal agenesis after in utero exposure to DBP at 500 (1/30 rats; 1/8 litters) and 750 DBP mg/kg/d (2/9 rats; 1/4 litters) when treatment was initiated at GD3, whereas, Mylchreest et al. (2000) reported no reproductive tract malformations in female offspring following in utero exposure to DBP beginning at GD12. Similarly, we observed reproductive tract malformations in female rats following DEHP exposure after implantation on GD8 (Gray et al., unpublished). Therefore, we hypothesized that the critical exposure period for reproductive effects may be slightly earlier in the female than in the male. To test this hypothesis, we evaluated a continuous dosing period of GD8–19, an early period (GD8–13) and a late period of GD14–19 (the critical exposure period for male reproductive malformations by phthalates).

The Mayer–Rokitansky–Kuster–Hauser (MRKH) syndrome in humans can be quite variable but is generally characterized by aplasia of the uterus and upper 2/3 of the vagina in a female with normal secondary sexual development and a 46, XX karyotype. The incidence of MRKH syndrome is estimated at 1/4000 to 1/5000 live female births (Sultan et al., 2009). Although familial cases of this syndrome suggest that it is transmitted in an autosomal dominant fashion with incomplete penetrance and variable expressivity, the specific genetic causes of the syndrome have not been identified. The term “phthalate syndrome” refers to the phthalate-induced reproductive malformations observed in male rodent offspring following exposure during the sexual differentiation period. We have observed that the female rat equivalent of the “phthalate syndrome” presents with many similarities to human MRKH syndrome. Therefore, the dose response and critical exposure period assessment data obtained in the current study will better define the female phthalate syndrome and thereby characterize a potential model for evaluating the genomic mechanism associated with MRKH syndrome.

## 2. Materials and methods

### 2.1. Animals

Adult female Sprague–Dawley (SD) rats (Charles River, Raleigh, NC) were mated by the supplier and shipped on gestation day 2 (GD2), with day of sperm plug positive = GD1. Animals were housed individually in 20 cm × 25 cm × 47 cm clear polycarbonate cages with laboratory-grade heat-treated pine shavings (Northeastern Products, Warrensburg, NY) with a 12:12 light/dark photoperiod (lights off at 19:00 h) at 20–24 °C. Pregnant and lactating females were fed Purina Rat Chow 5008 ad libitum. Animals were provided access to filtered (5 μm filter) municipal drinking water (Durham, NC) ad libitum. Water was tested monthly for *Pseudomonas* and every 4 months for a suite of chemicals including pesticides and heavy metals. The current study was conducted under protocols approved by the National Health and Environmental Effects Research Laboratory Institutional Animal Care and Use Committee in an Association for Assessment and Accreditation of Laboratory Animal Care-approved animal facility.

### 2.2. Doses and administration of chemicals

Laboratory-grade corn oil (CAS 8001-30-7, Cat# C-8627), and butyl benzyl phthalate (**BBP**; CAS 85-68-7, Cat# 308501, lot# 08523JQ, purity 98%); dibutyl phthalate (**DBP**; CAS 84-74-2, Cat# D-2270, lot# 109F0386, purity = 99%); di(2-ethylhexyl) phthalate (**DEHP**; CAS 117-81-7, Cat# P-6699, lot# 106H3487, purity = 99%); diisobutyl phthalate (**DiBP**; CAS 84-69-5, Cat# 152641, lot# 103141 C, purity = 99%), and dipentyl phthalate (**DPeP**; CAS 131-18-0, Cat# 80154, lot# 1151652, purity = 99%) were purchased from Sigma–Aldrich (St. Louis, MO). The doses were delivered in 2.5 μl corn oil per gram body weight.

#### 2.2.1. Study 1: 5-PE mixture dose response study

Pregnant rat dams were assigned to one of seven treatment groups (*n* = 6 dams/group) on GD8 in a manner that provided similar mean (±SE) body weight per treatment group prior to dosing. Sample sizes were based on power calculations performed on previous data demonstrating that 5–6 litters per dosage group was appropriate for detecting significant differences in incidence of malformations. Dams were dosed via oral gavage on GD8 through postnatal day (PND) 3 with 0, 65, 130, 260, 520, and 780 mg total phthalates/kg/d. The doses consisted of a mixture of five phthalates, including BBP, DBP, DEHP, DiBP and DPeP in ratio of 3:3:3:3:1, respectively. This ratio was formulated such that each phthalate contributed equally to the reduction in testicular testosterone production in fetal male rats exposed from GD8 to GD18 (Howdeshell et al., 2008).

#### 2.2.2. Study 2: 5-PE mixture critical period assessment

Dams were dosed by oral gavage as above with the mixture at 520 mg total phthalate/kg/d for one of 3 dosing periods (*n* = 5 dams per treatment group): GD8–19 (continuous), GD8–13 (early) or GD14–19 (late), repeating the second highest dosage level of the mixture from the above study which induced a high incidence of female reproductive tract malformations, but a lower level of perinatal mortality. A control group was dosed with corn oil from GD8–19 (*n* = 5 dams).

### 2.3. Neonatal endpoints

In both studies, control and treated dams were allowed to deliver naturally. On PND2 (day of birth = 1), the body weight and anogenital distance (AGD) of the pups (male and female) were measured as per Hotchkiss (Hotchkiss et al., 2004). Male offspring were assessed for areola or nipple retention on PND 14. All pups were weighed and weaned on PND22 and housed two to three per cage for maintenance until further analysis. Rat dams were euthanized after weaning the pups, their uteri were removed, and the number of uterine implants was recorded. Adult male offspring data will be published elsewhere (Howdeshell et al., in preparation).

### 2.4. Evaluation of female reproductive tract development and other tissue abnormalities

In the dose response study, two-thirds of the total numbers of female offspring (*n* = 101 females) were necropsied when they reached PND 77 (11 weeks of age). The remaining females (*n* = 65) were necropsied beginning at PND350 (50 weeks old). In the critical period assessment study, all female offspring were necropsied at PND 120 (17 weeks of age). Animals in both studies were examined for external (absence of vaginal opening) and internal reproductive malformations (uterine unicornis, complete absence of uterine horns, and hydrometrocolpos). Ovaries were excised and weighed. The animals were also examined for abnormalities of the kidney (hydronephrosis, kidney stones), blood in the bladder and anophthalmia, as reported previously, following DEHP in utero exposure (Narotsky and Kavlock, 1995). In cases of anophthalmia, the absence of the eyeball(s) was confirmed.

The various malformations were defined as follows:

*Absent vaginal opening (VO)*: no detected vagina orifice posterior to the urinary aperture.
*Uterine malformations*: unicornis (unilateral or bilateral), one or both of the uterine horns is absent or partially missing.
*Hydrometrocolpos*: accumulation of fluid in a uterine horn resulting in abnormal swelling.
*Hydronephrosis of the kidney(s)*: one or both kidneys contains signs of swelling or dilation of the kidney collection system, appears as agenesis of the renal medulla and can be mild (20–40% of medulla) or severe (>40% of medulla missing).
*Anophthalmia*: absence of one or both eyes.

### 2.5. Statistics

Data were analyzed by one-way analysis of variance (ANOVA) using the general linear measures procedures from the Statistical Analysis Systems (SAS, Inc., Cary, NC). Post hoc comparisons were made using the Least Squared Means procedure on SAS. Data were analyzed as litter means, with the exception of the malformation data which were analyzed both as individual means by Fishers exact test (Sigma Stat, Systat Software, San Jose, CA) and litter means using ANOVA. Data were considered significant at *p* < 0.05. AGD data were analyzed with and without body weight or the cube root of body weight as a covariate. Results were reported as AGD with no correction for body weight since there was no difference in outcome between the analyses with and without body weight as a covariate in Study 1 or 2 and neither covariate improved the error variance in the analysis.

## 3. Results

### 3.1. Study 1: 5-phthalate ester mixture dose response study

#### 3.1.1. Maternal and pregnancy data

**Fig. 1:**
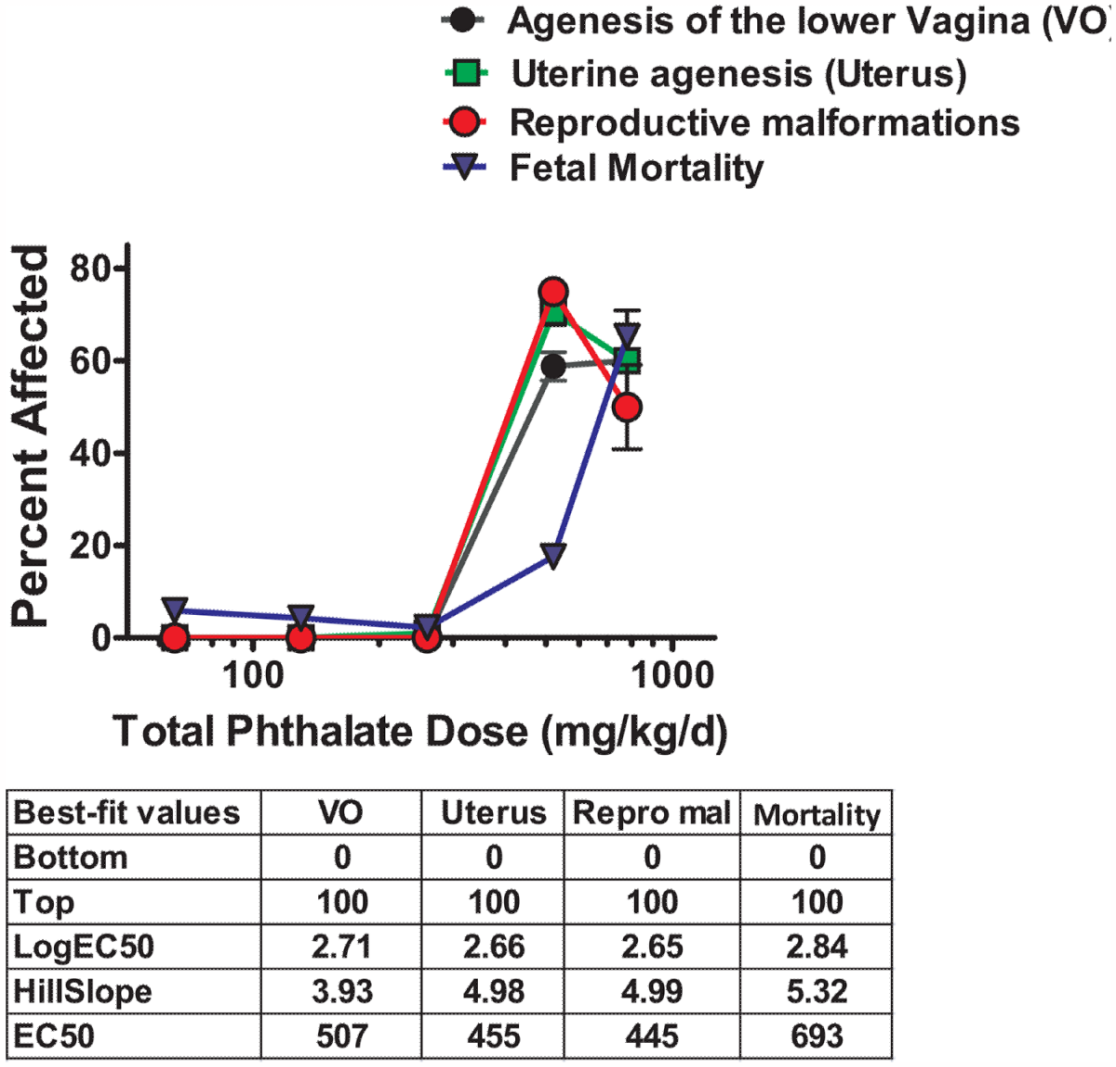
Malformations in female offspring and F1 pup mortality following GD8 through PND 3 administration of a dose range of a five phthalate mixture with the top dose of 780 mg total phthalates/kg/d, including 60 mg/kg/d DPP and 180 mg/kg/d of each of the following: BBP, DBP, DEHP, and DiBP. Each point represents the mean of 4–6 litters (±SEM).

The combination dose of five phthalates significantly reduced maternal body weight gain at the highest dose administered (780 mg/kg/d total phthalate; Table 1). The phthalate mixture treatment did not induce any overt toxicity or death in the dams. Administration of the five phthalate mixture significantly increased fetal mortality at a dose of 780 mg/kg/d total phthalates (which contains BBP, DBP, DEHP, and DiBP at 180 mg/kg/d per chemical and 60 mg DPP/kg/d; Table 1 and Fig. 1). While the total number of implantations was similar between treatments, the total number of fetuses was significantly decreased in the 780 mg/kg/d total phthalate dose and whole litter mid-pregnancy loss occurred in 2 of 6 dams at this dose of the phthalate mixture.

#### 3.1.2. Neonatal, infant and weaning data

Body weight of female pups on PND2 was significantly decreased by 780 mg/kg/d total phthalate dose, the highest dose of the phthalate mixture tested in this experiment. There were no treatment effects on AGD in female pups at PND2 (Table 1).

#### 3.1.3. Female reproductive tract development and other tissue abnormalities data

Administration of the phthalate mixture at dose of 520 and 780 mg total phthalate/kg/d significantly increased the incidence of reproductive tract malformations in the female offspring, including absence of a vaginal opening and uterine horn malformations (Fig. 1). The absence of a vaginal opening was significantly increased by the phthalate mixture at doses of 520 mg/kg/d (10/16 females; 3/4 litters; *p* < 0.001) and 780 mg/kg/d (3/5 females; 1/2 litters; *p* < 0.005). The incidence of uterine malformations was also increased with phthalate mixture doses of 520 mg/kg/d (12/16 females; 3/4 litters; *p* < 0.001) and 780 mg/kg/d (3/5 females; 1/2 litters; *p* < 0.005). The uterine abnormalities observed were: partial or complete agenesis of the uterine horns, and hydrometrocolpos. Most females with uterine malformations had only one uterine horn missing. The ovaries were present in all females and appeared grossly normal, regardless of treatment group. There were no reproductive malformations observed in female offspring of dams administered doses below 520 mg total phthalate/kg/d.

The incidence of anophthalmia in one or both eyes was significantly increased in the 780 mg total phthalate/kg/d dose group (2/5 females; 2/2 litters; *p* < 0.05) and occurred in 2/16 females (1/4 litters) of the 520 mg total phthalate/kg/d dose group (NS). In addition, one female in the 520 mg total phthalate/kg/d dose group was born without a tail (1/16 females). Neither body weight at necropsy or incidence of non-reproductive malformations was significantly affected by treatment. Unilateral hydronephrosis was observed in 4/16 females (2/4 litters) in the 520 mg total phthalate/kg/d and another female from this dose group lacked a right kidney; all females with kidney aberrations at the 520 mg total phthalate dose also had reproductive malformations. Bilateral hydronephrosis was observed in one female each of the control and 8.3 mg total phthalate/kg/d dose groups; both of these two females had normal vaginal openings.

### 3.2. Study 2: 5-phthalate ester mixture critical period assessment

#### 3.2.1. Maternal and pregnancy data

Administration of 520 mg total phthalate/kg/d significantly increased maternal body weight gain when dosing occurred from GD8 to 19 (Table 2). The mixture dose did not cause any overt toxicity in the dams; however one dam in the GD8–19 group exhibited complete litter loss. Overall, administration of the phthalate mixture from GD8–19 and GD8–13 significantly increased fetal mortality (Table 2). This dose level was slightly more toxic to pups than in Study 1.

#### 3.2.2. Neonatal data

The five phthalate mixture did not affect pup body weight in males or females on PND2 following any of the exposure periods. Female AGD was not affected by any dosing period, whereas male AGD was significantly decreased following administration of the mixture from GD8–19 and GD14–19 (Table 2). All female offspring retained 12 nipples. The mixture significantly increased male nipple retention in both the GD8–19 and GD14–19 dosing periods (Table 2).

#### 3.2.3. Female reproductive tract development and other tissue abnormalities data

**Fig. 2:**
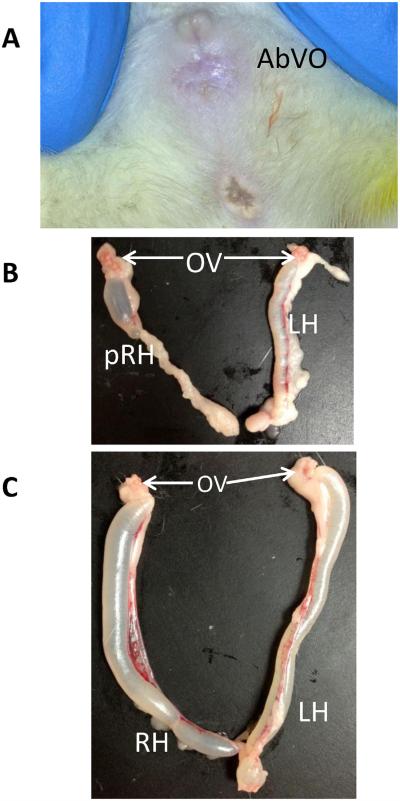
Examples of reproductive tract malformations detected in female offspring of dams administered 520 mg total phthalate/kg/d of a 5-phthalate mixture from GD8–19. (A) Absent vagina opening (AbVO) is characterized by no detected vagina orifice posterior to the urinary aperture. (B) Uterine unicornis on the right horn, characterized by absence of the posterior portion of the right horn (pRH = partial right horn). (C) Hydrometrocolpos is characterized by accumulation of fluid in a uterine horn resulting in abnormal swelling in both the right and left horns (RH, LH, respectively). Ovaries (OV) were present and appear normal in most females with reproductive tract malformations.

The incidence of female reproductive malformations in offspring of dams administered 520 mg total phthalate/kg/d was significantly increased when dosing occurred during GD8–19 (88%) and GD8–13 (22%), but not GD14–19 (0%; Table 3). The incidence of absent vaginal opening (Fig. 2a (*p* < 0.0001) and bilateral (*p* < 0.0001) and unilateral (*p* < 0.05) uterine agenesis were significantly increased in the continuous dosing period group only (GD8–19). One female in the GD8–19 had had partial agenesis of the right uterine horn with the remainder fluid-filled (Fig. 2b) and one complete hydrometrocolpos (Fig. 2c). One female with complete agenesis of both uterine horns had ovaries that failed to descend below the kidneys. The ovaries in all remaining females from all dosage groups appeared grossly normal and there was no significant difference in paired ovary weights between groups. There were no reproductive malformations observed in female offspring of dams administered the phthalate mixture during GD14–19 (Table 3).

One female in the GD8–19 group had anophthalmia in both eyes and one female in the GD8–13 group had anophthalmia in one eye (Table 3). Hydronephrosis in one or both kidneys was observed in all phthalate-treated dosing period groups and was significantly increased from control in the GD14–19 group (Table 3, *p* < 0.05).

## 4. Discussion

Although less studied than in males, phthalate esters are also reproductive toxicants in the fetal and pregnant female rat. In the current study, we report high incidences of reproductive malformations (including: hydrometrocolpos, agenesis or complete aplasia of the uterus, and vaginal opening and canal agenesis) in the adult female rat following in utero exposure to a mixture of five PEs. We did not fully identify the critical exposure period for inducing these malformations in the female. We do provide evidence to support the hypothesis that the female window is earlier in gestation than the male window which is encompassed by GD14–19 since a low incidence of female reproductive tract malformations were induced with GD8–13 exposure but not GD14–18. In contrast to the female reproductive effects and the fetal mortality, the effects on the perinatal male offspring in study 2 were restricted to those exposed during GD14–19 (reduced AGD and retained nipples).

This is the first report demonstrating a high incidence of female reproductive malformations following gestational exposure to a mixture of phthalates. Previous studies focusing on single phthalate exposures report relatively lower incidences of female effects. A combination of in utero and lactational exposure to DBP (250 mg/kg/d) caused reduced fecundity and induced a low incidence of uterine malformations (partial agenesis or absence of implants in one horn) in female rats, while their male siblings exhibited a low incidence of hypospadias, undescended testes and lowered epididymal sperm counts (Gray et al., 1999). Low frequencies of delayed vaginal opening and partial to complete agenesis of the uterus have also been reported for DBP at 500 and 750 mg/kg/d (Mylchreest et al., 1998). Thus, reproductive tract malformations reportedly occur in both males and females at about the same phthalate ester doses, however they occur less frequently in the female. The five phthalate mixture dose response data for females reported in this study produced an ED50 of 536.4 mg total phthalate/kg/d, which is approximately 2-fold higher than the ED50 for males (265.4 mg total phthalate/kg/d with a similar curve slope) (Howdeshell et al., in preparation). These data support the hypothesis that females are less sensitive than males to the reproductive toxicity of phthalates.

Female rats exposed to phthalate esters from puberty through pregnancy and lactation also show altered reproductive physiology, but no gross reproductive malformations were reported. Female Long Evans (LE) rats orally dosed with DBP (250–1000 mg/kg/d from weaning through pregnancy/lactation) cycled and mated normally (with untreated males), however several females exposed to 500 mg DBP/kg/d aborted their litters around mid-pregnancy, and treatment with 1000 mg/kg/d DBP resulted in complete infertility. In a follow up study, female LE rats treated with DBP (500 and 1000 mg/kg/d from weaning through pregnancy/lactation through several pregnancies) had significantly decreased litter size associated with reduced serum progesterone levels and ovarian progesterone production on GD13 (Gray et al., 2006). In addition, several of the DBP-treated dams had hemorrhagic corpora lutea. Increased post-implantation embryo loss and inhibited uterine decidualization have also been reported in Wistar rats treated with DBP (750–1500 mg/kg/d from GD0 to 8 (Ema et al., 2000)). In a non-human primate, chronic DEHP exposure (500 and 2500 mg/kg/d) from weaning to adulthood led to increased ovarian weights in juvenile common marmoset females (Tomonari et al., 2006). The enlarged ovaries had large corpus lutea, which is a common characteristic of older common marmoset females and is suggestive of accelerated aging with DEHP treatment (Tomonari et al., 2006). These data together suggest that one mechanism of action of phthalate esters on the pubertal and adult female rat is the inhibition of steroidogenesis, which may subsequently impair the maintenance of pregnancy.

On the contrary, the results of the current study lead us to hypothesize that the five phthalate mixture administered during gestational development in the current study is likely operating through earlier gene-related events. If the fetal ovaries were “masculinized” by the phthalate mixture and produced androgens then we would expect to see agenesis of the lower vagina and nipples with no effect on the uterus. However we did not see agenesis of the nipples at 14 days of age. If the fetal ovaries were fully “masculinized” and secreted both anti-Mullerian hormone (AMH) and testosterone then this would cause agenesis of the lower vagina, the utero-cervix complex, and the nipples. Nevertheless, we detected a combination of uterine and vaginal malformations without nipple agenesis. In addition, we observed that both “ovaries” in one treated female from the second experiment were located above the kidneys. Histological confirmation of these tissues as ovarian tissue is in progress. The reproductive malformations seen in the females exposed in utero to the mixture of phthalates (uterus and vagina) do not fully conform to those expected from in utero exposure to excessive androgen and AMH levels (which would include the nipples, uterus and vagina). In that case, it appears that masculinization of fetal ovarian hormone production by the phthalates is not likely the primary or sole mechanism responsible for the malformations detected in the current study.

**Fig. 3:**
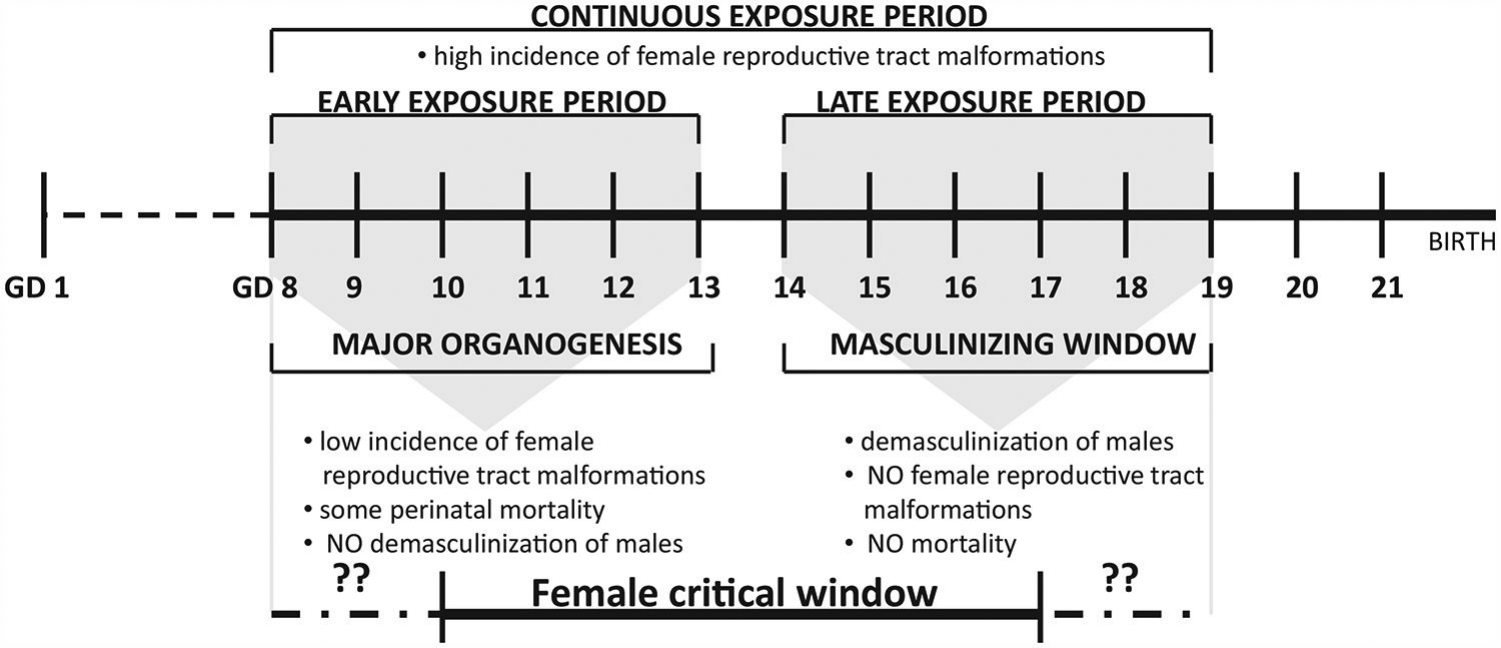
Dosing periods and critical window effects following administration of a phthalate mixture (520 mg total phthalate/kg/d) to pregnant dams for one of three different exposure periods (illustrated above the gestational day (GD) timeline). Endpoint observations are listed below the GD timeline for effects observed in both the early and late exposure periods. Based on these results, the female critical period appears to span both the early and late periods.

Identification of the most critically vulnerable period of development to PEs in the fetal female rat would facilitate investigation of the gene changes associated with the detected malformations. In males, GD16–18 is the period where differentiation to the male phenotype is most sensitive to phthalate disruption (Carruthers and Foster, 2005). Indeed, we demonstrate in the current study that male demasculinization (retained nipples at PND 14, reduced AGD at PND 2) occurs when exposure take place from GD14–19 or GD8–19 but not when exposure is earlier (GD8–13). On the contrary, we did observe neonate mortality (equivalent rates in males and females) in both the early (GD8–13) and continuous (GD8–19) dosing groups, but not in the late (GD14–19) dosing group. This finding clearly delineates the critical period of development leading to male reproductive malformations from that leading to neonatal mortality. While there was no evidence of demasculinization of males from the early period, we did detect female reproductive malformations when dosing occurred during this period. The incidence of female effects in the early period was lower than that of the continuous period (which was 88%), suggesting that our dosing periods did not encompass the entire critical window. Based on the partial incidence of malformations in the early period and higher percent incidence in the continuous period, further investigation of the female critical period should span across both the early and later periods (Fig. 3).

This report highlights the striking similarity of the “female phthalate syndrome” to MRKH syndrome in women, including uterine and vaginal agenesis and renal defects. To date, investigations into the etiology of MRKH syndrome have been unsuccessful in identifying the genetic components of the disease (Morcel et al., 2007; Sultan et al., 2009). Nevertheless, there is no clear association of mutations in these genes and the incidence of MRKH in humans (Bernardini et al., 2009; Cheroki et al., 2006). In spite of the historic difficulty in determining the genetic etiology of MRKH syndrome, we have herein identified a potentially useful model for understanding this disease. This would not be the first case in which a chemically induced malformation in the rodent has served as a useful model for determining the mechanism of development of a poorly understood congenital malformation in humans. For example, Ostby et al. (1985), Gray et al. (1983) and Wickman et al. (1993) identified diaphragmatic hernias in rat and mouse pups following in utero exposure to the now banned herbicide nitrofen. Since the time of this discovery, several hundred published studies have utilized this animal model to investigate the molecular events disrupted by nitrofen during development of diaphragmatic hernias and hypoplasia of the lung in order to better understand events that may be occurring in children in utero that display these lesions spontaneously. In another example, monosodium glutamate is routinely used to induce hypothalamic lesions in neonatal rats as model for understanding obesity and diabetes mechanisms in humans (Diemen et al., 2006; Mello et al., 2001; Takahashi et al., 2006; Sukhanov et al., 1999). These examples further support the concept that phthalate-induced female reproductive tract malformations may serve as a model for MRKH syndrome in humans, pending additional investigation.

Until now, the major focus of phthalate toxicity has been the male anti-androgenic and reproductive toxicity properties, particularly in fetal-exposed offspring. The results presented in this study draw attention to the need to concurrently evaluate female reproductive toxicity of PEs. Not only is this important from the standpoint of phthalate human risk assessment, but as discussed here, further characterization of this model of MRKH syndrome may potentially make great strides toward elucidating the genetic origins of the disease, which may have a resounding impact on the relatively high frequency of afflicted women.

## Figures and Tables

**Table 1:** Maternal body weight gain, number of implantations, litter size at postnatal day (PND) 2, and fetal mortality, as well as female offspring body weight and anogenital distance, of SD rat dams administered a dose range of a combination dose of five phthalates from gestation day (GD) 8 to PND3. The top dose used here of the combination dose was administered at 780 mg of the five phthalates/kg/d, including 60 mg/kg/d DPP and 180 mg/kg/d of each of the following: BBP, DBP, DEHP, and DiBP.

Dose as total phthalate (mg/kg/d)	0	65	130	260	520	780
No. of dams on GD18	6	5	6	6	5	6
No. of dams with whole litter loss	0	0	0	0	0	2
Maternal body wt. at GD8 (g)	259.8 ± 10.2	265.3 ± 7.6	269.2 ± 6.4	270.6 ± 5.7	270.8 ± 8.5	272.1 ± 7.0
Maternal body wt. at GD20 (g)	364.1 ± 7.7	361.7 ± 7.5	381.1 ± 11.7	361.2 ± 6.4	385.4 ± 13.4	325.6 ± 16.5
Maternal body wt. gain during pregnancy (g)^a^	104.3 ± 5.1	96.4 ± 5.3	111.9 ± 5.8	90.6 ± 3.8	114.6 ± 19.3	**53.5 ± 13.3** c
No. of implantations	14.0 ± 0.5	12.2 ± 1.4	14.6 ± 0.6	14.2 ± 0.9	14.6 ± 0.9	12.8 ± 0.9
No. of live pups on PND2	12.5 ± 1.3	11.4 ± 1.2	14.0 ± 0.4	13.8 ± 0.8	12.2 ± 1.6	**4.3 ± 1.8** c
Fetal mortality (%)^b^	11.7 ± 6.6	5.9 ± 2.6	4.3 ± 2.0	2.2 ± 1.4	17.6 ± 5.6	**65.3 ± 13.0** c
Body wt. of female pups on PND2 (g)	7.4 ± 0.3 (33, 6)^d^	7.5 ± 0.4 (24, 5)	7.3 ± 0.2 (35, 6)	7.0 ± 0.2 (41, 6)	7.0 ± 0.3 (35, 5)	**5.7 ± 0.6**^c^ **(11, 4)**
AGD of female pups on PND2 (mm)	1.59 ± 0.03 (33, 6)	1.53 ± 0.07 (24, 5)	1.62 ± 0.04 (35, 6)	1.70 ± 0.04 (41, 6)	1.70 ± 0.06 (35, 5)	1.61 ± 0.15 (11, 4)

a Maternal body wt gain during pregnancy = body wt. at GD20–body wt. at GD8.

b Fetal mortality = (no. of implantations – no. oflive pups on PND2)/no. of implantations.

c *p* < 0.05 versus control (shown in bold).

d Numbers in parenthesis indicate that number of individuals and litters per treatment group.

**Table 2:** Maternal body weight gain, number of implantations, litter size at postnatal day (PND) 2, and fetal mortality, as well as female offspring body weight and anogenital distance, of SD rat dams administered 520 mg/kg/d of a mixture of 5 PEs (including 40 mg/kg/d DPP and 120 mg/kg/d of each of the following: BBP, DBP, DEHP, and DiBP), over varying dosing period lengths.

Dosing period (gestational days; GD)	Control	8–19	8–13	14–19
No. of dams on GD19	5	4	5	5
No. of dams with whole litter loss	0	1	0	0
Maternal body wt. at beginning of dosing GD8 or GD14 (g)	(GD8) 257.9 ± 3.5 (GD14) 299.2 ± 5.0	250.4 ± 2.2	258.2 ± 1.7	313.4 ± 5.5
Maternal body wt. at GD13 or GD19 (g)	(GD13) 290.0 ± 4.9 (GD19) 348.6 ± 4.2	**314.3 ± 6.0** c	284.4 ± 5.9	360.66 ± 10.3
Maternal body wt. gain during pregnancy (g)^a^	(GD8–19) 90.7 ± 3.7 (GD8–13) 32.1 ± 2.5 (GD14–19) 49.5 ± 1.8	**63.6 ± 6.0** c	26.2 ± 5.5	47.3 ± 6.9
No. of implantations	13.8 ± 0.9	13.2 ± 0.4	12.6 ± 1.1	14.0 ± 0.6
No. of live pups on PND2	12.6 ± 0.93	**4.2 ± 1.74** c	**8.0 ± 1.14** c	13.2 ± 0.8
Fetal mortality (%)^b^	8.3 ± 5.3	**70.7 ± 12.5** c	**37.4 ± 10.6** c	5.9 ± 2.9
Body wt. of female pups on PND2 (g)	7.53 ± 0.12 (33,5)	7.19 ± 0.30 (8,3)	7.43 ± 0.26 (12,4)	7.80 ± 0.11 (31,5)
AGD of female pups on PND2 (mm)	1.59 ± 0.04 (33,5)	1.53 ± 0.09 (8,3)	1.51 ± 0.04 (12,4)	1.54 ± 0.03 (31,5)
Body wt. of male pups on PND2 (g)	8.04 ± 0.15 (30,5)	7.68 ± 0.27 (13,3)	7.59 ± 0.14 (28,5)	7.94 ± 0.07 (35,5)
AGD of male pups on PND2 (mm)	3.46 ± 0.07 (30,5)	**2.48 ± 0.89**^c^ (13,3)	3.46 ± 0.06 (28,5)	**2.51 ± 0.06**^c^ (35,5)
Male nipple retention (# out of 12 possible)	0 ± 0 (28,5)	**9.5 ± 1.1**^c^ (13,4)	0.5 ± 0.5 (24,5)	**11.8 ± 0.3**^c^ (22,4)

a Maternal body wt gain during pregnancy = body wt. at GD20–body wt. at GD8.

b Fetal mortality = (no. of implantations – no. of live pups on PND2)/no. of implantations.

c *p* < 0.05 versus control (shown in bold).

**Table 3:** Female SD rat offspring postnatal malformations following in utero exposure of varying dosing period lengths to 520 mg/kg/d of a mixture of 5 PEs (including 40 mg/kg/d DPP and 120 mg/kg/d of each of the following: BBP, DBP, DEHP, and DiBP).

Dosing period (gestational days; GD)	Control	8–19	8–13	14–19
Number of individuals, number of litters	11,5	8,4	9,3	24,5
Body weight at necropsy (g)	243.0 ± 5.9	230.4 ± 3.8	239.5 ± 6.3	245.1 ± 4.6
Reproductive malformation (% incidence)^a^	0.0 ± 0.0	**87.5 ± 12.5** b	**22.2 ± 14.6** b	0.0 ± 0.0
Absent VO (% incidence)	0.0 ± 0.0	**75.0 ± 16.4** b	11.1 ± 11.1	0.0 ± 0.0
Uterine agenesis total (% incidence)	0.0 ± 0.0	**75.0 ± 16.4** b	22.2 ± 14.7	0.0 ± 0.0
Bilateral (% incidence)	0.0 ± 0.0	**50.0 ± 18.9** b	11.1 ± 11.1	0.0 ± 0.0
Unilateral (% incidence)	0.0 ± 0.0	**25.0 ± 16.4** b	11.1 ± 11.1	0.0 ± 0.0
Hydrometrocolpos (% incidence)	0.0 ± 0.0	12.5 ± 12.5	0.0 ± 0.0	0.0 ± 0.0
Paired ovarian weight (mg)	126.9 ± 6.6	106.7 ± 12.3	131.7 ± 4.3	121.6 ± 8.2
Hydronephrosis (% incidence)	0.0 ± 0.0	12.5 ± 12.5	22.2 ± 14.6	**29.2 ± 9.5** b
Anopthalmia (% incidence)	0.0 ± 0.0	12.5 ± 12.5	11.1 ± 11.1	0.0 ± 0.0

a Reproductive malformation = absent VO + uterine agenesis + hydrometrocolpus

b *p* < 0.05 versus control (shown in bold).

## HIGHLIGHTS

- A chemically induced animal model of the human MRKH syndrome.
- A phthalate syndrome in female rats: uterine and vaginal agenesis.
- A phthalate mixture disrupts male and female rat sexual differentiation.
- Differences in critical periods for disrupted sex differentiation and mortality.
